# A call for action to improve access to care and treatment for patients with rare diseases in the Asia-Pacific region

**DOI:** 10.1186/s13023-014-0137-1

**Published:** 2014-09-16

**Authors:** Swee-Sung Soon, Gilberto Lopes, Hwee-Yong Lim, Durhane Wong-Rieger, Salmah Bahri, Lucy Hickinbotham, Anand Jha, Bor-Sheng Ko, Diana MacDonell, Jasmine Roah-Fang Pwu, Ruby Shih, Ekaphop Sirachainan, Dong-Churl Suh, Janet Wale, Xiao Zhang, Hwee-Lin Wee

**Affiliations:** Department of Pharmacy, National University of Singapore, 18 Science Drive 4, 117543 Singapore, Singapore; Johns Hopkins University School of Medicine, 733 North Broadway, 21205 Baltimore, MD USA; Novena Cancer Centre, Mount Elizabeth Novena Specialist Centre, 38 Irrawaddy Road, 329563 Singapore, Singapore; Institute for Optimizing Health Outcomes, Toronto, Canada; Pharmaceutical Services Division, Ministry of Health, Block E1, E3 , E6, E7 & E10, Parcel E, Federal Government Administration Centre, 62590 Putrajaya, Malaysia; Novartis Oncology, 20 Pasir Panjang Road, #10-25/28 Mapletree Business City, 117439 Singapore, Singapore; National Taiwan University Hospital, No.7, Zhongshan S. Road, Zhongzheng District, Taipei City, 10002 Taiwan; Pharmaceutical Benefits Advisory, Bedford Park, Australia; Center for Drug Evaluation 1 F, No.15-1, Sec.1, Hangjou S. Road, Taipei, Taiwan; Division of Medical Review and Pharmaceutical Benefits, Bureau of National Health Insurance, No.140, Sec.3, Hsinyi Road, Taipei, 10634 Taiwan; Department of Medicine, Ramathibodi Hospital, 34/1 Soi Saengsuksa School, Isarapap Road, Min Buri, Bangkok 10510 Thailand; College of Pharmacy, Chung-Ang University, 221 Heukseok-dong, Dongjak-gu, Seoul, South Korea; HTAi Interest Group for Patient/Citizen Involvement in Health Technology Assessment, Melbourne, Australia; The Public Health School, Southeast University, 2 Sipailou Rd, 210096 Nanjing, China

**Keywords:** Challenges, Policy, Access to care, Asia-Pacific region

## Abstract

This article is a call for action to the relevant stakeholders to improve access to care and treatment for patients with rare diseases in the Asia-Pacific region by looking into three main areas: (a) developing legislative definitions to confer enforceable protection, (b) creating or strengthening policies by objectively measuring the impact brought about by rare diseases and establishing platforms to reach out to the rare disease community, and (c) fostering collaboration across sectors and countries. It is hoped that these suggested actions can catalyze discussions and progress in the region.

## Letters to the editor

Dear Editor,

Despite many healthcare improvements in the Asia-Pacific region, patients with rare diseases are still falling through the cracks. Many healthcare systems tend to consider each rare disease no differently from other more common diseases although these patients face a greater risk of being marginalized [1,2]. Based on the World Health Organization’s prevalence estimate, collectively, rare diseases can affect 292 million people in the Asia-Pacific region [3].

Faced with uneven access to care and treatment of rare diseases, we wish to highlight (a) legal framework, (b) healthcare policy, and (c) collaborations as the three areas where rare disease patients in the region can benefit most. We are aware that separate considerations may be required for rare diseases that are treatable versus those that are not. However, for this letter, we would like to make a collective call for action for all types of rare diseases.

Developing legal definitions for terms such as “orphan drugs” and “rare disease” is pivotal to setting up legal frameworks to protect the well-being of the rare disease community. Looking to Taiwan, the Rare Disease Control and Orphan Drug Act brings significant economic relief to patients with genetic rare diseases through subsidies or reimbursement [4]. Despite the power that legislation wields, the long and arduous journey towards passing a bill can prompt one to look for non-legislative alternatives. Thailand stands out in the region because even without a legislative framework for rare diseases or orphan drugs, working definitions culminated in the inclusion of the Orphan Drug List into the National Drug List in 2012 [5]. While the lack of a legislative framework could still achieve some operative success, only the law can confer enforceable protection uniformly across the country, thus avoiding abrupt policy changes or postcode lottery situations.

Policy-wise, reaching out and understanding the rare disease community are primary steps to identifying inadequacies in existing systems. In Europe, the European Organisation for Rare Diseases (EURORDIS) led a survey program that lent many insights to the predicament of the rare disease community [6], and has important implications in policy formulation. Information-gathering initiatives like this should be considered in the Asia-Pacific region. The European Union Committee of Experts on Rare Diseases (EUCERD) later established with a mandate to formulate and implement activities in rare diseases [7] illustrates how a multi-national cross-sector panel can organize and implement rare disease initiatives. A ‘EUCERD’-equivalent for the region can raise the awareness and commitment of policy-makers to act. To reach out to the community, the Taiwan Foundation for Rare Disorders represents patients with rare diseases in Taiwan before the policy-makers [8], showcasing a model suitable for smaller countries; in bigger countries, the National Organization for Rare Disorders presents another model as an alliance of individual patient groups [9]. Pooling previously fragmented groups on a national level can generate the much-needed awareness to overcome the lack of political will.

For implementation, a coordinated and pragmatic approach should be considered. As tackling rare diseases is resource-intensive, concerted efforts should be channeled to meet a need without duplication. To this, several European countries have launched defined national strategies on rare diseases [10]. Drawing from Europe’s experience, the European Commission had been a vital catalyst in elevating awareness and coordinating information exchanges. These brought visibility to the rare disease community on the policy table. The high-level approach taken by the European Commission has galvanized policy-level support and a similar approach should be considered in the Asia-Pacific region. Having a taskforce to champion this cause will be a useful starting point.

Collaboration is key to advancing care for patients with rare diseases. Funded by a technology grant, South Korea’s Rare Disease Knowledge Base showcases successful inter-agency collaboration by linking genetic, clinical and administrative databases to mitigate misdiagnosis. To be acquainted with global developments in rare diseases, platforms such as the Global Rare Disease Patient Registry and Data Repository (GRDR) and the International Rare Disease Research Consortium (IRDiRC) can be explored. Countries stand to benefit from GRDR’s existing data elements and open-source patient registry templates [11]. To facilitate the generation and exchange of data, countries in the region should actively contribute to existing registries [12], or consider starting registries for diseases unique to the region. On the research front, the IRDiRC links researchers and organizations investing in rare diseases research [13]. Of the 36 IRDiRC member organizations, only four were from the Asia-Pacific region, highlighting opportunities for the region to step up especially for diseases unique to the region.

In conclusion, we have identified three areas that warrant attention and action from relevant stakeholders to improve access to care and treatment for patients with rare diseases in the Asia-Pacific region. Several initiatives offer good examples to emulate but progress across the region is variable and more can be done through developing legislative definitions, strengthening rare disease policies, and fostering stronger collaboration.

## Abbreviations

EUCERD: European union committee of experts on rare diseases
EURORDIS: European Organisation for Rare Diseases
GRDR: Global rare disease patient registry and data repository
IRDiRC: International rare disease research consortium
